# Prognostic factors in hospitalized community-acquired pneumonia: a retrospective study of a prospective observational cohort

**DOI:** 10.1186/s12890-017-0424-4

**Published:** 2017-05-02

**Authors:** Akihiro Ito, Tadashi Ishida, Hironobu Tokumasu, Yasuyoshi Washio, Akio Yamazaki, Yuhei Ito, Hiromasa Tachibana

**Affiliations:** 1 Department of Respiratory Medicine, Ohara Memorial Kurashiki Healthcare Foundation, Kurashiki Central Hospital, Miwa 1-1-1, Kurashiki, Okayama 710-8602 Japan; 2 Department of Clinical Research Institute, Ohara Memorial Kurashiki Healthcare Foundation, Kurashiki Central Hospital, Miwa 1-1-1, Kurashiki, Okayama 710-8602 Japan; 3 Department of Respiratory Medicine, National Hospital Organization Minami Kyoto Hospital, Nakaashihara11, Joyo, Kyoto 610-0113 Japan

**Keywords:** Azithromycin, Combination therapy, Community-acquired pneumonia, Prognosis, Severity score

## Abstract

**Background:**

To date, only few studies have examined the prognostic factors of community-acquired pneumonia (CAP) defined according to the latest criteria, which excludes healthcare-associated pneumonia (HCAP). Therefore, we aimed to investigate the factors that affect prognosis, and evaluate the usefulness of existing pneumonia severity scores for predicting the prognosis of CAP.

**Methods:**

We retrospectively analyzed patients with CAP, excluding HCAP, who were enrolled prospectively between April 2007 and February 2016. Four patients who used macrolides other than azithromycin (AZM) were excluded. We used age, sex, comorbidities, laboratory findings and antimicrobial therapy as prognostic variables. The primary outcome was 30-day mortality and secondary outcome was ICU admission. We also performed receiver operating characteristic curve analysis of Pneumonia Severity Index (PSI), Infectious Diseases Society of America (IDSA)/American Thoracic Society (ATS) severe criteria, CURB-65 and A-DROP pneumonia severity scores.

**Results:**

Among 1834 CAP patients, mean age was 73.5 ± 14.3 years; 1281 (69.8%) were men; and 30-day mortality was 6.7% (122/1834). In total, 1830 patients were analyzed. Multivariate analysis identified age [Odds Ratio (OR): 1.04, 95% Confidence Interval (CI): 1.02–1.07], chronic obstructive pulmonary disease (COPD) [OR: 1.77, 95% CI: 1.13–2.76], malignancy (OR: 2.25, 95% CI: 1.25–4.06), body temperature (OR: 0.81, 95% CI: 0.67–0.99), respiratory rate (OR: 1.04, 95% CI: 1.01–1.07), PaO_2_/FiO_2_ ≤ 250 (OR: 3.15, 95% CI: 1.93–5.14), Alb (OR: 0.27, 95% CI: 0.19–0.39), BUN (OR: 1.01, 95% CI: 1.00–1.02), and mechanical ventilation (OR: 2.99, 95% CI: 1.75–5.12) as prognostic factors. AZM and β-lactam combination therapy significantly reduced 30-day mortality (OR: 0.50, 95% CI: 0.26–0.97). Areas under the curve of PSI, IDSA/ATS severe criteria, CURB-65 and A-DROP were 0.759, 0.746, 0.754 and 0.764, respectively.

**Conclusions:**

Increasing age, presence of COPD and malignancy as comorbidities, hypothermia, tachypnea, PaO_2_/FiO_2_ ratio ≤250 mmHg, low Alb level, high BUN level and mechanical ventilatory support predict a worse prognosis; AZM combination therapy should be considered for CAP, excluding HCAP. All four pneumonia severity scores are useful for assessing the severity of CAP defined by the latest criteria.

**Trial registration:**

UMIN-CTR UMIN000004353.﻿ Registered 7 October 2010. Retrospectively registered.

**Electronic supplementary material:**

The online version of this article (doi:10.1186/s12890-017-0424-4) contains supplementary material, which is available to authorized users.

## Background

Worldwide, several severity scoring systems have been used to guide decisions on the site of care and to assess the prognosis of community-acquired pneumonia (CAP). Examples of such scoring systems are the Pneumonia Severity Index (PSI) [1], severe pneumonia criteria by the Infectious Diseases Society of America (IDSA)/American Thoracic Society (ATS) (IDSA/ATS severe pneumonia criteria) [2], CURB-65 by the British Thoracic Society [3] and A-DROP by the Japanese Respiratory Society [4]. In the 2005 IDSA/ATS guidelines [5], healthcare-associated pneumonia (HCAP) was defined as being associated with a greater risk of antimicrobial-resistant infections and worse prognosis than CAP.

Although these pneumonia severity scoring indices incorporate a variety of prognostic factors for CAP, including patient characteristics, such as age, sex, comorbidities, vital signs and laboratory findings, the prognostic factors are distinct from severity scoring indices. All these pneumonia severity scores mentioned above were established before the definition of HCAP was proposed. Although many reports have investigated the prognostic factors and utility of these scoring systems for assessing the severity of CAP, only few have evaluated them in terms of CAP defined by the latest criteria, which excludes HCAP. In this study, we aimed to investigate the prognostic factors of CAP defined by the latest criteria, including not only patient characteristics, vital signs and laboratory findings, but also initial antibiotic therapy as prognostic factors; we also evaluated whether pre-existing pneumonia severity scoring systems are useful for predicting prognosis in CAP excluding HCAP.

## Methods

### Study design and setting

This study retrospectively analyzed hospitalized CAP patients who were enrolled in a prospective, observational, cohort study at Kurashiki Central Hospital between April 2007 and February 2016. CAP was diagnosed in accordance with the IDSA/ATS guidelines as [2]: presence of at least one of the clinical symptoms of cough, sputum, fever, dyspnea, and pleuritic chest pain, plus at least more than one finding of coarse crackles on auscultation or elevated inflammatory biomarkers, in addition to a new infiltrate on chest radiography. We enrolled consecutively hospitalized patients diagnosed with pneumonia in this cohort. Exclusion criteria were: age <15 years, acquired immune deficiency syndrome, hospital-acquired pneumonia, and HCAP [5]. This study was performed as part of a clinical study for pneumonia (UMIN000004353) after October 2010 and was approved by the institutional review board of Kurashiki Central Hospital (approval number 2235). All patients gave their informed consent to participate in this study.

In all the patients, severity of pneumonia was assessed on admission with the use of PSI [1], IDSA/ATS severe pneumonia criteria [2], CURB-65 score [confusion, urea >7 mmol/L, respiratory rate ≥30 breaths per minute, low blood pressure (systolic <90 mmHg or diastolic ≤60 mmHg), and age ≥65 y] [3] and A-DROP score [age ≥70 years in men or age ≥75 years in women, blood urea nitrogen ≥21 mg · dL^−1^ or dehydration, oxyhemoglobin saturation measured by pulse oximetry ≤90% or partial pressure of oxygen in arterial blood ≤60 mmHg, confusion, or systolic blood pressure ≤90 mmHg] [4].

All patients received antimicrobial agents at the discretion of the physician in charge and in accordance with the recommendations of the CAP guidelines of the Japanese Respiratory Society [4]. Blood tests and chest X-ray images were examined to assess the effectiveness of the antimicrobials. Basically, patients were treated in the intensive care unit (ICU) if they needed mechanical ventilatory support and/or vasopressor drugs.

### Microbiological investigations

As far as possible, we tried to obtain samples of sputum and blood for cultures at the time of admission, and blood for measuring serum antibodies to detect the causative pathogens of CAP. A bacterial cause was identified if the following criteria were met: (1) positive sputum culture of more than 1+ on a qualitative test or 10^5^ on a quantitative test, with reference to the sputum Gram stain; (2) positive blood culture (excluding contaminated normal skin flora); (3) positive pleural fluid culture; (4) positive urinary antigen test for *Streptococcus pneumoniae* and *Legionella pneumophila*; (5) seroconversion or four-fold increase in antibodies for *Mycoplasma pneumoniae* and *Chlamydophila pneumoniae*; and (6) ≥1:320 on a single antibody test for *M. pneumoniae* PA antibody (FUJIREBIO, Tokyo, Japan) or a cut-off index of ≥2.0 on a *C. pneumoniae* IgM antibody test using the Hitazyme® assay (Hitachi Chemical, Tokyo, Japan).

### Prognostic variables

In this study, we assessed age, sex, smoking status, comorbidities, vital signs and laboratory findings that influenced prognosis. Comorbidities included chronic heart disease, chronic obstructive pulmonary disease (COPD), diabetes mellitus, cerebrovascular disease, malignant disease, chronic kidney disease, and chronic liver disease. We diagnosed COPD using the Global Initiative for chronic obstructive lung disease (GOLD) definition [6], and patients who were already diagnosed and treated as COPD in other hospitals and had emphysema on chest tomography were also included. We defined malignant disease as one that was active at the time of admission or was diagnosed within one year of admission.

We did not evaluate disturbances in consciousness as a prognostic variable in this study, because it is included as a factor in severity scoring systems and because we did not assess consciousness separately in all patients. Also, we used a partial pressure of arterial oxygen/fraction of inspired oxygen (PaO_2_/FiO_2_) ratio of ≤250 mmHg as a surrogate indicator of oxygenation, since we did not perform arterial blood gas analysis in all patients. We estimated PaO_2_ from oxyhemoglobin saturation measured by pulse oximetry.

Previous reports have shown that CAP patients with bacteremia [7] or multilobar pneumonia [8] have a worse prognosis; therefore, we included these variables in the analysis.

For the treatment of CAP, some systematic reviews and meta-analyses of observational cohort studies have shown that combination therapy of macrolides and β-lactams (BLs) improve prognosis [9, 10]; however, this was opposed by two randomized controlled studies [11, 12]. In this study, we investigated the efficacy of macrolide combination therapy, fluoroquinolone (FQN) combination therapy, and BL monotherapy in terms of 30-day mortality.

The primary study outcome assessed was 30-day mortality and secondary outcome was direct admission to the ICU at the time of hospitalization. We also evaluated the usefulness of existing pneumonia severity scores, such as PSI, IDSA/ATS severe pneumonia criteria, CURB-65 and A-DROP, for predicting 30-day mortality in CAP excluding HCAP. We designated patients who were discharged within 30 days of admission and those who did not follow-up after discharge as survivors.

### Statistical analysis

Continuous variables were expressed as mean and standard deviation (SD), whereas categorical variables were expressed as frequency (percentage), as appropriate. Continuous variables were analyzed by Student’s t-test and categorical variables were assessed with the chi-square test. Univariate analysis was performed for predicting 30-day mortality. Multivariate analysis using stepwise logistic regression analysis was conducted for all variables that were found to have a *P* value of ≤0.1 on univariate analysis. For antimicrobial therapy, azithromycin (AZM) was used in almost all patients who received macrolide combination therapy, except in 4 patients who were given clarithromycin (*n* = 3) and erythromycin (*n* = 1). Therefore, we excluded these 4 patients and analyzed macrolide combination therapy as AZM combination therapy. To assess the usefulness of existing pneumonia severity scores for predicting the prognosis of CAP, we performed Receiver Operating Characteristic (ROC) curve analysis with application of the Bonferroni correction for two-way comparisons of the AUCs of pneumonia severity scores. All statistical tests were two-tailed, and we considered a *P* value <0.05 as significant. Analyses were performed using R (version 3.0.3, Vienna, Austria).

## Results

### Patient characteristics

The baseline characteristics of the 1834 patients enrolled in this prospective cohort study are listed in Table 1. Males comprised about 70% of the study population. The most common comorbidity was chronic heart disease (30.2%), followed by COPD (24.0%). The 30-day mortality rate was 6.7% (122/1834).

**Table 1:** Characteristics of patients with community-acquired pneumonia

	*n* (%)
All patients	1834
Male	1281 (69.8)
Age (y)	73.5 ± 14.3
Smoking status ^a^	
Current	292 (15.9)
Past	850 (46.3)
Never	692 (37.7)
Comorbidity	
Chronic heart disease	554 (30.2)
COPD^b^	440 (24.0)
Diabetes mellitus	364 (19.8)
Cerebrovascular disease	354 (19.3)
Malignancy^c^	167 (9.1)
Chronic kidney disease	136 (7.4)
Chronic liver disease	111 (6.1)
Bacteremia	93 (5.1)
Duration of hospitalization (days)	15.4 ± 12.7
ICU admission	95 (5.2)
A-DROP (score)	
0	206 (11.2)
1	486 (26.5)
2	575 (31.4)
3	426 (23.2)
4	126 (6.9)
5	15 (0.8)
CURB-65 (score)	
0	193 (10.5)
1	554 (30.2)
2	634 (34.6)
3	329 (17.9)
4	106 (5.8)
5	18 (1.0)
PSI (score)	103.4 ± 34.2
PSI (class)	
I	37 (2.0)
II	211 (11.5)
III	456 (24.9)
IV	799 (43.6)
V	331 (18.0)
IDSA/ATS severe criteria	
Yes	585 (31.9)
No	1249 (68.1)
In-hospital mortality	132 (7.2)
30-day mortality	122(6.7)

Data are presented as mean ± SD or *n* (%)

*Abbreviations: A-DROP* age ≥70 years in men or age ≥75 years in women, blood urea nitrogen ≥21 mg · dL^−1^ or dehydration, oxyhemoglobin saturation measured by pulse oximetry ≤90% or partial pressure of oxygen in arterial blood ≤60 mmHg, confusion or systolic blood pressure ≤90 mmHg, *ATS* American Thoracic Society, *COPD* chronic obstructive pulmonary disease, *CURB-65* confusion, urea >7 mmol/L, respiratory rate ≥30 breaths/min, low blood pressure (systolic <90 mmHg or diastolic ≤60 mmHg), and age ≥65 years, *ICU* intensive care unit, *IDSA* Infectious Diseases Society of America, *PSI* Pneumonia Severity Index

^a^Current: Patients who are current smokers and have been smoking more than 100 cigarettes in their entire life, Past: Patients who quit smoking more than a month ago and have been smoking more than 100 cigarettes in their entire life, Never: Patients who have not smoked in the past month and have not smoked more than 100 cigarettes in their entire life

^b^We diagnosed COPD using the GOLD definition [6]. Patients who were already diagnosed and treated as COPD in other hospitals and had emphysema on chest tomography were also included

^c^This included malignant disease that was active at the time of admission or was diagnosed within one year of admission

### Etiology of community-acquired pneumonia

In this study, causative pathogens were identified in 854 patients (46.6%); of them, 107 were infected with more than two microorganisms. Of the 1834 patients, we performed sputum tests in 1652 (90.1%), blood culture in 1756 (95.7%), and urinary antigen tests in 1691 (92.2%) patients. Additional file 1: Table S1 shows the distribution of the causative pathogens identified by these tests. In seven patients, performance of these tests was not feasible. There were 107 patients with multiple bacterial etiologies; therefore, the cumulative infection rate was over 100%. The most common causative microorganism was *S. pneumoniae* (22.2%), followed by *Haemophilus influenzae* (7.0%). The prevalence of multidrug-resistant pathogens was 2.4%; these included *Pseudomonas aeruginosa* (1.8%), extended-spectrum *β*-lactamase (ESBL) producing *Escherichia coli* (0.1%), and methicillin-resistant *Staphylococcus aureus* (0.5%).

### Initial antibiotic therapy

Table 2 shows the initial antimicrobial agents in all patients. Of the 1248 patients on monotherapy, 1181 patients (94.6%) were treated with BLs. Three hundred seventy-seven patients received AZM and BLs combination therapy; AZM was given orally at 500 mg/day for 3 days in 182 patients, as a single dose of 2 g/day in 159 patients, and intravenously at 500 mg/day in 32 patients. In patients on FQN combination therapy with BLs (*n* = 119), pazufloxacin (73.1%) was most prescribed, followed by levofloxacin (22.7%).

**Table 2:** Initial antibiotic agents in all patients with community-acquired pneumonia

	*n* = 1834				
	Monotherapy	Combination therapy			
	*n* = 1248	*n* = 586			
		Macrolides^a^	FQN^b^	TC	LCM
		*n* = 377	*n* = 119	*n* = 82	*n* = 8
Penicillins	933	199	47	61	2
Cephalosporins	223	163	23	19	6
Carbapenems	25	7	41	2	0
Fluoroquinolones	49	8	―	0	0
Macrolides	12	―	8	0	0
Tetracycline	3	0	0	―	0
Lincomycin	1	0	0	0	―
Oseltamivir	2	0	0	0	0

*Abbreviations: FQN* fluoroquinolone, *TC* tetracycline, *LCM* lincomycin

^a^Oral azithromycin (500 mg/day) for 3 days in 182, oral azithromycin (2 g/day) single dose in 159, azithromycin (500 mg/day) injection in 32, oral erythromycin in 1, and oral clarithromycin in 3 patients

^b^Ciprofloxacin in 4, tosufloxacin in 1, pazufloxacin in 87, and levofloxacin in 27 patients

### Prognostic factors for 30-day mortality in community-acquired pneumonia

The characteristics of survivors and non-survivors are shown in Table 3. In univariate analysis, age, COPD and malignancy as comorbidities, vital signs such as body temperature, heart rate, respiratory rate and PaO_2_/FiO_2_ ≤ 250, laboratory findings such as albumin (Alb), blood urea nitrogen (BUN), creatinine (Cr), hematocrit (Ht) and C-reactive protein (CRP) levels, multilobar pneumonia, bacteremia, AZM or FQN combination therapy, and treatment including mechanical ventilatory and vasopressor drug support were significant prognostic factors in patients hospitalized with CAP. In multivariate analysis, age [Odds Ratio (OR): 1.04, 95% Confidence Interval (CI): 1.02–1.07], COPD [OR: 1.77, 95% CI: 1.13–2.76], malignant disease (OR: 2.25, 95% CI: 1.25–4.06), body temperature (OR: 0.81, 95% CI: 0.67–0.99), respiratory rate (OR: 1.04, 95% CI: 1.01–1.07), PaO_2_/FiO_2_ ≤ 250 (OR: 3.15, 95% CI: 1.93–5.14), Alb (OR: 0.27, 95% CI: 0.19–0.39), BUN (OR: 1.01, 95% CI: 1.00–1.02), AZM combination therapy (OR: 0.50, 95% CI: 0.26–0.97) and mechanical ventilation (OR: 2.99, 95% CI: 1.75–5.12) were significant prognostic factors for CAP (Table 4).

**Table 3:** Characteristics of community-acquired pneumonia survivors and non-survivors

	Survivors	Non-survivors	Univariate
	*n* = 1708	*n* = 122	*P* value
Male	1187 (69.5)	92 (75.4)	0.20
Age (y)	73.2 ± 14.4	78.8 ± 10.4	<0.001
Smoking status^a^			0.26
Current	273 (16.0)	19 (15.6)	
Past	780 (45.7)	68 (55.7)	
Never	655 (38.3)	35 (28.7)	
Comorbidity			
Chronic heart disease	509 (29.8)	45 (36.9)	0.12
COPD^b^	393 (23.0)	46 (37.7)	<0.001
Diabetes mellitus	340 (19.9)	24 (19.7)	1.00
Cerebrovascular disease	325 (19.0)	27 (22.1)	0.47
Malignancy	148 (8.7)	19 (15.6)	0.02
Chronic kidney disease	125 (7.3)	11 (9.0)	0.47
Chronic liver disease	100 (5.9)	10 (8.2)	0.39
Vital signs			
Body temperature (°C)	37.9 ± 1.1	37.4 ± 1.2	<0.001
Systolic blood pressure (mmHg)	129 ± 26	126 ± 30	0.19
Heart rate (beats/min)	98 ± 19	103 ± 25	0.004
Respiratory rate (breaths/min)	23 ± 7	28 ± 9	<0.001
PaO_2_/FiO_2_ ≤ 250 (mmHg)^c^	554 (32.4)	92 (75.4)	<0.001
Laboratory examinations			
Alb (g/dL)	3.2 ± 0.6	2.6 ± 0.6	<0.001
BUN (mg/dL)	21.8 ± 14.5	34.5 ± 22.2	<0.001
Cr (mg/dL)	1.01 ± 0.8	1.27 ± 1.2	0.001
Na (mmol/L)	136.7 ± 4.2	137.2 ± 6.3	0.25
Ht (%)	37.1 ± 5.5	35.6 ± 6.5	0.004
Plt (×10^4^/μL)	22.5 ± 10.2	22.8 ± 10.2	0.75
WBC (×10^3^/μL)	12.3 ± 6.2	12.1 ± 6.4	0.75
CRP (mg/L)	125 ± 94	164 ± 108	<0.001
Multilobar pneumonia	939 (55.0)	99 (81.1)	<0.001
Bacteremia	73 (4.3)	20 (16.4)	<0.001
β-lactam monotherapy	1109 (64.9)	72 (59.0)	0.22
AZM combination	354 (20.7)	12 (9.8)	0.005
FQN combination	81 (4.7)	30 (24.6)	<0.001
MINO combination	79 (4.6)	3 (2.5)	0.37
Mechanical ventilation	99 (5.8)	40 (32.8)	<0.001
Vasopressor drug usage	60 (3.5)	26 (21.3)	<0.001
ICU admission	67 (3.9)	28 (23.0)	<0.001
A-DROP (score)			<0.001
0	204 (11.9)	1 (0.8)	
1	478 (28.0)	7 (5.7)	
2	545 (31.9)	28 (23.0)	
3	372 (21.8)	54 (44.3)	
4	97 (5.7)	29 (23.8)	
5	12 (0.7)	3 (2.5)	
CURB-65 (score)			<0.001
0	192 (11.2)	0 (0)	
1	540 (31.6)	12 (9.8)	
2	594 (34.8)	39 (32.0)	
3	294 (17.2)	35 (28.7)	
4	74 (4.3)	32 (26.2)	
5	14 (0.8)	4 (3.3)	
PSI (score)	100.7 ± 32.3	140.9 ± 37.2	<0.001
PSI (class)			<0.001
I	36 (2.1)	0 (0)	
II	210 (12.3)	1 (0.8)	
III	447 (26.2)	8 (6.6)	
IV	750 (43.9)	47 (38.5)	
V	265 (15.5)	66 (54.1)	
IDSA/ATS severe criteria			<0.001
Yes	490 (28.7)	95 (77.9)	
No	1218 (71.3)	27 (22.1)	

Data are presented as mean ± SD or *n* (%)

*Abbreviations: A-DROP* age ≥70 years in men or age ≥75 years in women, blood urea nitrogen ≥21 mg · dL^−1^ or dehydration, oxyhemoglobin saturation measured by pulse oximetry ≤90% or partial pressure of oxygen in arterial blood ≤60 mmHg, confusion, or systolic blood pressure ≤90 mmHg, *Alb* Albumin, *ATS* American Thoracic Society, *AZM* azithromycin, *BUN* Blood urea nitrogen, *COPD* chronic obstructive pulmonary disease, *Cr* Creatinine, *CRP* C-reactive protein, *CURB-65* confusion, urea >7 mmol/L, respiratory rate ≥30 breaths/min, low blood pressure (systolic <90 mmHg or diastolic ≤60 mmHg), and age ≥65 years, *FiO*_*2*_ fraction of inspired oxygen, *FQN* fluoroquinolone, *Ht* Hematocrit, *ICU* intensive care unit, *IDSA* Infectious Diseases Society of America, *MINO* minomycin, *Na* Sodium, *PaO*_*2*_ partial pressure of arterial oxygen, *Plt* Platelet, *PSI* Pneumonia Severity Index, *WBC* white blood cell

^a^Current: Patients who are current smokers and have been smoking more than 100 cigarettes in their entire life, Past: Patients who quit smoking more than a month ago and have been smoking more than 100 cigarettes in their entire life, Never: Patients who have not smoked in the past month and have not smoked more than 100 cigarettes in their entire life.

^b^We diagnosed COPD according to the GOLD definition [6]. Patients who were already diagnosed and treated as COPD in other hospitals and had emphysema on chest tomography were also included.

^c^Arterial blood gas analyses were not performed in 513 of the survivors and 12 of the non-survivors; in these patients, arterial oxygen partial pressure was estimated from oxyhemoglobin saturation measured by pulse oximetry.

**Table 4:** Univariate and multivariate analysis of prognostic factors for 30-day mortality

	Univariate			Multivariate		
Prognostic factors	OR	95% CI	*P* value	OR	95% CI	*P* value
Age	1.04	1.02–1.06	<0.001	1.04	1.02–1.07	<0.001
COPD	2.03	1.38–2.97	<0.001	1.77	1.13–2.76	0.01
Malignancy	1.94	1.16–3.26	0.01	2.25	1.25–4.06	0.007
Body temperature (°C)	0.65	0.55–0.77	<0.001	0.81	0.67–0.99	0.045
Heart rate (beats/min)	1.01	1.00–1.02	0.004			
Respiratory rate (breaths/min)	1.08	1.06–1.11	<0.001	1.04	1.01–1.07	0.008
PaO_2_/FiO_2_ ≤ 250 (mmHg)	6.39	4.18–9.76	<0.001	3.15	1.93–5.14	<0.001
Alb (g/dL)	0.22	0.16–0.30	<0.001	0.27	0.19–0.39	<0.001
BUN (mg/dL)	1.03	1.02–1.04	<0.001	1.01	1.00–1.02	0.04
Cr (mg/dL)	1.27	1.10–1.47	0.001			
Ht (%)	0.95	0.92–0.99	0.004			
CRP (mg/L)	1.04	1.02–1.06	<0.001			
Multilobar pneumonia	3.53	2.22–5.60	<0.001			
Bacteremia	4.39	2.58–7.49	<0.001			
AZM combination	0.42	0.23–0.77	0.005	0.50	0.26–0.97	0.04
FQN combination	6.55	4.10–10.5	<0.001			
Mechanical ventilation	7.93	5.16–12.2	<0.001	2.99	1.75–5.12	<0.001
Vasopressor drug usage	7.44	4.49–12.3	<0.001			

*Abbreviations: Alb* Albumin, *AZM* azithromycin, *BUN* Blood urea nitrogen, *CI* confidence interval, *COPD* chronic obstructive pulmonary disease, *Cr* Creatinine; *CRP* C-reactive protein, *FiO*_*2*_ fraction of inspired oxygen, *FQN* fluoroquinolone, *Ht* Hematocrit, *OR* odds ratio, *PaO*_*2*_ partial pressure of arterial oxygen

### Predictive factors for ICU admission in community-acquired pneumonia

In univariate analysis, vital signs such as body temperature, heart rate, systolic blood pressure, respiratory rate and PaO_2_/FiO_2_ ≤ 250, laboratory findings such as Alb, BUN, Cr and CRP value, multilobar pneumonia, bacteremia and treatment with mechanical ventilation and vasopressor drugs were significant predictive factors for ICU admission on the day of hospitalization. Of these, body temperature (OR: 0.62, 95% CI: 0.47–0.81, *P* < 0.001), respiratory rate (OR: 1.05, 95% CI: 1.00–1.09, *P* = 0.04), PaO_2_/FiO_2_ ≤ 250 (OR: 9.34, 95% CI: 3.62–24.1, *P* < 0.001), CRP (OR: 1.04, 95% CI: 1.01–1.07, *P* = 0.009), mechanical ventilation (OR: 18.4, 95% CI: 8.65–39.1, *P* < 0.001) and vasopressor drug usage (OR: 7.40, 95% CI: 3.29–16.6, *P* < 0.001) were significant predictive factors in multivariate analysis.

### Usefulness of existing pneumonia severity scores for predicting 30-day mortality

**Fig. 1:**
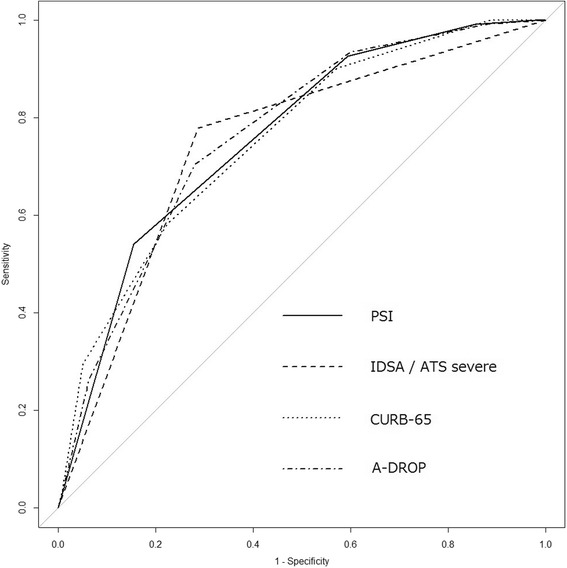
ROC curve analysis of ability of pneumonia severity scores to predict 30-day mortality in cases of community acquired pneumonia (CAP), excluding healthcare-associated pneumonia (HCAP). The AUCs of PSI, IDSA/ATS severe pneumonia criteria, CURB-65 and A-DROP were 0.759 (95% CI: 0.721–0.796), 0.746 (95% CI: 0.707–0.784), 0.754 (95% CI: 0.713–0.794) and 0.764 (95% CI: 0.726–0.802), respectively. In comparisons of the AUCs of these four pneumonia severity scores with each other, the *P* value was 1.0 in all comparison groups adjusted by the Bonferroni method (PSI vs. IDSA/ATS severe pneumonia criteria, PSI vs. CURB-65, PSI vs. A-DROP, IDSA/ATS severe pneumonia criteria vs. CURB-65, IDSA/ATS severe pneumonia criteria vs. A-DROP, and CURB-65 vs. A-DROP). A-DROP, age ≥70 years in men or age ≥75 years in women, blood urea nitrogen ≥21 mg · dL^−1^ or dehydration, oxyhemoglobin saturation measured by pulse oximetry ≤90% or partial pressure of oxygen in arterial blood ≤60 Torr, confusion, or systolic blood pressure ≤90 mmHg; ATS, American Thoracic Society; AUC, Area under the curve; CURB-65: confusion, urea >7 mmol/L, respiratory rate ≥30 breaths/min, low blood pressure (systolic <90 mmHg or diastolic ≤60 mmHg), and age ≥65 years; IDSA, Infectious Diseases Society of America; PSI, Pneumonia Severity Index; ROC, Receiver Operating Characteristic

The values of areas under the curve (AUC) in ROC analysis for prediction of 30-day mortality were 0.759 (95% CI: 0.721–0.796), 0.746 (95% CI: 0.707–0.784), 0.754 (95% CI: 0.713–0.794) and 0.764 (95% CI: 0.726–0.802) for PSI class, IDSA/ATS severe pneumonia criteria, CURB-65 and A-DROP scores, respectively (Fig. 1). There were no significant differences in the predictive ability of each pneumonia severity score.

## Discussion

In this study, we showed that increasing age, presence of COPD and malignancy as comorbidities, low body temperature, tachypnea, PaO_2_/FiO_2_ ≤ 250, low Alb levels, high BUN levels and the need for mechanical ventilatory support were predictors of a poor prognosis in CAP patients. We also found that AZM combination therapy with BLs was a predictor of good prognosis, and that the existing pneumonia severity indices have a good predictive ability for the prognosis of CAP. The results of this study are important because the study population comprised patients who were diagnosed as CAP based on the new criteria, which excludes those with HCAP.

All four pneumonia severity scores, including PSI [1], IDSA/ATS severe pneumonia criteria [2], CURB-65 [3] and A-DROP [4] were previously shown to be useful for predicting prognosis in patients diagnosed with CAP based on the old criteria, which includes HCAP patients. Although some previous reports have investigated the prognostic factors and usefulness of existing pneumonia severity scores in predicting the prognosis of CAP [13, 14], our report is valuable because patient vital signs and antibiotic therapy, especially macrolide combination therapy, were not evaluated in previous studies.

Age is included as a prognostic factor in the PSI [1], CURB-65 [3] and A-DROP scoring systems [4]. Our study showed that increasing age is also an important prognostic factor for CAP defined according to the latest criteria, which exclude HCAP. As for the comorbidities, Restrepo et al reported that CAP patients with COPD showed significantly higher 30-day mortality (HR 1.32; 95% CI 1.01–1.74) and 90-day mortality (HR 1.34; 95% CI 1.02–1.76) than those without COPD [15]. Molinos et al also showed that COPD was an independent poor prognostic factor in CAP, after adjusting for age (OR 2.62; 95% CI 1.08–6.39) [16]. In this study, we showed similar results. Therefore, our study underscores the importance of pneumococcal vaccination in the prevention of pneumonia in COPD patients, as recommended by the Global Initiative for Chronic Obstructive Lung Disease for stable COPD [6]. In fact, Maruyama et al [17] reported in their study that the 23-valent pneumococcal vaccine significantly reduced pneumococcal pneumonia by 63.8% (95% CI 32.1–80.7; *P* = 0.0015) and all-cause pneumonia by 44.8% (95% CI 22.4–60.8; *P* = 0.0006). Malignancy is also a known prognostic factor for CAP, with a score of 30 points in PSI [1]. Tashiro and colleagues reported that the presence of malignancy is a poor prognostic factor for CAP in patients aged 18–64 years [18]. We also showed that malignancy is a prognostic factor for CAP in patients aged over 15 years.

All four pneumonia severity scores include vital signs as prognostic factors, although they are different from pneumonia severity indices. Respiratory status is included in all pneumonia severity scores and respiratory rate is adopted in CURB-65 [3], while respiratory failure is adopted in PSI [1], IDSA/ATS severe pneumonia criteria [2] and A-DROP [4]. A PaO_2_/FiO_2_ ratio of ≤250 is one of the prognostic factors in IDSA/ATS severe pneumonia criteria. Our study showed that both tachypnea and PaO_2_/FiO_2_ ≤ 250 are poor prognostic factors. Hypothermia is adopted as a prognostic factor in PSI (body temperature less than 35 °C or more than 40 °C, 15 points) and IDSA/ATS severe pneumonia criteria (body temperature less than 36 °C); our study showed similar results. However, since the cut-off values of these factors for predicting prognosis are unknown, further studies are needed to determine these.

Regarding laboratory findings, our data indicated that Alb and BUN were poor prognostic factors of CAP. BUN is included in all four pneumonia severity scores, while Alb is not included. Previous reports showed that low levels of Alb are a poor prognostic factor in CAP [19, 20] and in both CAP and HCAP [21]. Our study also supported these findings, which suggests that, in future, Alb should be included as a prognostic factor in existing pneumonia severity indices.

Recently, some systematic reviews and meta-analysis indicated that compared with BL monotherapy, macrolide combination therapy reduced CAP mortality rate [9, 10]. However, the studies included in these reviews were all observational in design. On the other hand, two randomized controlled trials [11, 12] did not demonstrate a reduced mortality rate with macrolide combination therapy. Therefore, the efficacy of macrolide combination therapy in reducing mortality in CAP is controversial. Previous reports that assessed the usefulness of macrolide therapy in patients with CAP, including some cases of HCAP, included erythromycin, clarithromycin, and AZM. In this study, we showed that AZM combination therapy with BLs reduced mortality rate in CAP patients, excluding HCAP patients.

Macrolides, including AZM, have anti-inflammatory properties and immunomodulating effects, such as regulation of neutrophil chemotaxis, decreased pro-inflammatory cytokine production, and regulation of adhesion molecule expression [22, 23]. AZM demonstrated anti-inflammatory and antivirulent characteristics in mouse and human studies on *P. aeruginosa* [24, 25]. An experimental study on pneumococcal pneumonia in mice showed that AZM combination therapy with ampicillin was effective in downregulating lung inflammation and accelerating bacterial clearance [26]. These effects of AZM, in addition to its antibacterial properties, may have brought about the reduction of mortality in CAP.

In systematic reviews and meta-analyses, the AUC of summary ROC curves for predicting 30-day mortality with PSI and CURB-65 in CAP were reported as 0.81 and 0.80, respectively [27]. Shindo et al. [28] reported that A-DROP was as useful in assessing the severity of CAP as CURB-65, and their AUCs for predicting 30-day mortality were 0.846 and 0.835, respectively. Compared to previous reports, the results of our study indicated mild low AUCs for all pneumonia severity scores.

However, since the AUC was about 0.75 for each scoring system, we believe that the existing pneumonia severity scores are useful for predicting the prognosis of CAP defined by recent criteria.

This study has certain limitations. First, since it was performed at a single center, the applicability of our results to other areas or countries is uncertain. Regardless, the present study analyzed over 1800 CAP patients, which is a large number of patients. Second, our study had some missing data. We did not include disturbance of consciousness as a prognostic variable because we could not assess it in all patients as a separate item. Although arterial blood gas analysis was not performed in 513 patients from among the survivors and 12 patients from among non-survivors, oxyhemoglobin saturation, as measured by pulse oximetry, was ≥90% in almost all these patients. Hence, we estimated that the PaO_2_/FiO_2_ ratio in these patients was at least >250. Analysis of disturbance of consciousness and PaO_2_/FiO_2_ ratio as separate prognostic factors might have revealed different results. Finally, although AZM combination therapy was associated with a good prognosis in CAP, the best formulation for a particular population is unclear. In this study, 91.6% of patients received AZM in the oral form; therefore, oral AZM may be sufficient, at least for its anti-inflammatory effect. However, it is important to note that use of AZM combination therapy in all CAP patients may increase antimicrobial resistance and cost. Therefore, the CAP population that would benefit from AZM combination therapy should be determined in future randomized controlled trials.

## Conclusions

Age, COPD and malignant disease as comorbidities, hypothermia, tachypnea, PaO_2_/FiO_2_ ≤ 250, low Alb level, high BUN level and treatment including mechanical ventilation are poor prognostic factors for CAP defined by the new criteria. All existing pneumonia severity scores, such as PSI, IDSA/ATS severe pneumonia criteria, CURB-65 and A-DROP are useful in predicting the prognosis of patients with CAP, excluding HCAP. AZM combination therapy with BLs reduced the 30-day mortality across all severities of CAP. Although AZM seemed to be a good choice of therapy, it is important to determine the population who will best benefit from it, while minimizing antibiotic resistance and high treatment cost.

## Additional file

Additional file 1: Table S1. Distribution of causative microorganisms of community-acquired pneumonia. (DOCX 14 kb)

## Abbreviations

Alb: Albumin
ATS: American Thoracic Society
AZM: Azithromycin
BLs: β-lactams
BUN: Blood urea nitrogen
CAP: Community-acquired pneumonia
CI: Confidence interval
COPD: Chronic obstructive pulmonary disease
Cr: creatInine
CRP: C-reactive protein
FiO_2_: Fraction of inspired oxygen
FQN: Fluoroquinolone
HCAP: Healthcare-associated pneumonia
Ht: Hematocrit
ICU: Intensive care unit
IDSA: Infectious diseases society of America
OR: Odds ratio
PaO_2_: Partial pressure of arterial oxygen
PSI: Pneumonia severity index
