# Supplementary material for: Prognostic factors in hospitalized community-acquired pneumonia: a retrospective study of a prospective observational cohort
Source: BMC Pulm Med. 2017 May 2;17:78. doi: 10.1186/s12890-017-0424-4 (PMC5414343; doi:10.1186/s12890-017-0424-4)
Supplement: Additional file 1: Table S1. — Distribution of causative microorganisms of community-acquired pneumonia. (DOCX 14 kb) [file 12890_2017_424_MOESM1_ESM.docx]

Table S1. Distribution of causative microorganisms of community-acquired pneumonia

|  | N (%)  N = 1827^a^ |
| --- | --- |
| *Streptococcus pneumoniae* | 406 (22.2) |
| *Haemophilus influenzae* | 128 (7.0) |
| β-lactamase-negative, ampicillin-resistant | 28 (1.5) |
| *Moraxella catarrhalis* | 57 (3.1) |
| *Chlamydophila pneumoniae* | 52 (2.8) |
| *Streptococcus anginosus* | 46 (2.5) |
| Methicillin-susceptible *Staphylococcus aureus* | 37 (2.0) |
| *Pseudomonas aeruginosa* | 33 (1.8) |
| *Klebsiella pneumoniae* | 29 (1.6) |
| *Streptococcus* spp. | 27 (1.5) |
| *Legionella pneumophila* | 27 (1.5) |
| Anaerobes | 23 (1.3) |
| *Mycoplasma pneumoniae* | 23 (1.3) |
| *Escherichia coli* | 10 (0.5) |
| Extended-spectrum β-lactamase-producing | 2 (0.1) |
| Methicillin-resistant *Staphylococcus aureus* | 9 (0.5) |
| Influenza virus | 6 (0.3) |
| Other gram-negative rods ^b^ | 5 (0.3) |
| Other pathogens ^c^ | 5 (0.3) |
| *Chlamydophila psittaci* | 4 (0.2) |
| *Enterobacter aerogenes* | 3 (0.2) |
| Unknown | 980 (53.6) |

^a^ Excluding 7 patients in whom no test was performed. There were 107 patients with multiple etiologies; therefore, the sum of the infection rates is over 100%.

^b^ *Acinetobacter spp*: 2, *Flavobacterium* spp: 1, *Klebsiella oxytoca*: 1, *Proteus mirabilis*: 1, *Peptoniphilus asaccharolyticus*: 1

^c^ *Corynebacterium spp*: 2, *Actinomyces spp*: 1, *Kocuria kristinae*: 1, Measles virus: 1
